# Postpartum mood among universally screened high and low socioeconomic status patients during COVID-19 social restrictions in New York City

**DOI:** 10.1038/s41598-020-79564-9

**Published:** 2020-12-24

**Authors:** Michael E. Silverman, Laudy Burgos, Zoe I. Rodriguez, Omara Afzal, Alyssa Kalishman, Francesco Callipari, Yvon Pena, Ruth Gabay, Holly Loudon

**Affiliations:** 1 Department of Psychiatry, Icahn School of Medicine at Mount Sinai, The Mount Sinai Hospital, New York, USA; 2 MICDS, St. Louis, USA

**Keywords:** Psychology, Environmental social sciences, Health care, Risk factors

## Abstract

The mental health effects of the Severe Acute Respiratory Syndrome Coronavirus 2 (SARS-CoV-2) and the Coronavirus Disease 2019 (COVID-19) pandemic on postpartum women are of increasing concern among mental health practitioners. To date, only a handful of studies have explored the emotional impact of the pandemic surrounding pregnancy and none have investigated the consequence of pandemic-related social restrictions on the postpartum mood of those living among different socioeconomic status (SES). All postpartum patients appearing to the Mount Sinai Health System for their postpartum appointment between January 2, 2020 and June 30, 2020, corresponding to before and during pandemic imposed social restrictions, were screened for mood symptomatology using the Edinburgh Postnatal Depression Scale (EPDS). Each patient’s socioeconomic status (high/low) was determined by their location of clinical service. A total of 516 postpartum patients were screened. While no differences in EPDS scores were observed by SES prior to social restrictions (U = 7956.0, z = − 1.05, *p* = .293), a significant change in mood symptomatology was observed following COVID-19 restrictions (U = 4895.0, z = − 3.48, *p* < .001), with patients living in lower SES reporting significantly less depression symptomatology (U = 9209.0, z = − 4.56, *p* < .001). There was no change in symptomatology among patients of higher SES (U = 4045.5, z = − 1.06, *p* = .288). Postpartum depression, the most common complication of childbearing, is a prevalent, cross-cultural disorder with significant morbidity. The observed differences in postpartum mood between patients of different SES in the context of temporarily imposed COVID-19-related social restrictions present a unique opportunity to better understand the specific health and social support needs of postpartum patients living in urban economic poverty. Given that maternal mental illness has negative long-term developmental implications for the offspring and that poor mental health reinforces the poverty cycle, future health policy specifically directed towards supporting postpartum women living in low SES by ameliorating some of the early maternal burdens associated with balancing employment-family-childcare demands may assist in interrupting this cycle while simultaneously improving the long-term outcomes of their offspring.

## Introduction

Symptoms associated with depression are extremely common following childbirth^1^, with a considerable number of postpartum patients being diagnosed with clinically significant depression^2^. Because early postpartum depression can result in negative personal, family, and child developmental outcomes^3^, it is a serious public health concern. Given that postpartum patients and their newborn offspring represent physically, psychologically, and potentially sociologically vulnerable populations^4,5^ and in light of the worldwide impact of the Coronavirus Disease 2019 (COVID-19) pandemic, the health of those who have recently given birth has been a focus of increasing concern among obstetricians, pediatricians, mental health clinicians, Centers for Disease Control, and policymakers^6,7^.

The relationship between socioeconomic status (SES) and health, the income gradient—the relative increase in mortality with lower income—accounts for a greater loss of health than any risk factor other than normal human aging^8^. While questions remain concerning the causal direction of mental health and poverty^9,10^, that is, whether poverty increases the risk of mental health problems or whether poverty is a consequence of ill mental health, observations have repeatedly suggested that minority women living in low SES are among the most vulnerable for postpartum mood disruption^11,12^. Indeed, differences in postpartum health care following delivery, including suboptimal treatment^13^, distrust of providers^14^, racism^15^, religious factors^16^, perceived negative caregiver traits^17^, and poor health literacy^18^ are likely partially responsible for these observations. Similarly, social and economic factors such as unavailable or inadequate childcare^19^, limited partner and social support^20,21^, and reduced flexibility of available time secondary to financial obligations including both formal and informal employment^22,23^ likely also play a role in contributing to poorer postpartum physical and mental health outcomes among those living in lower SES.

**Figure 1:**
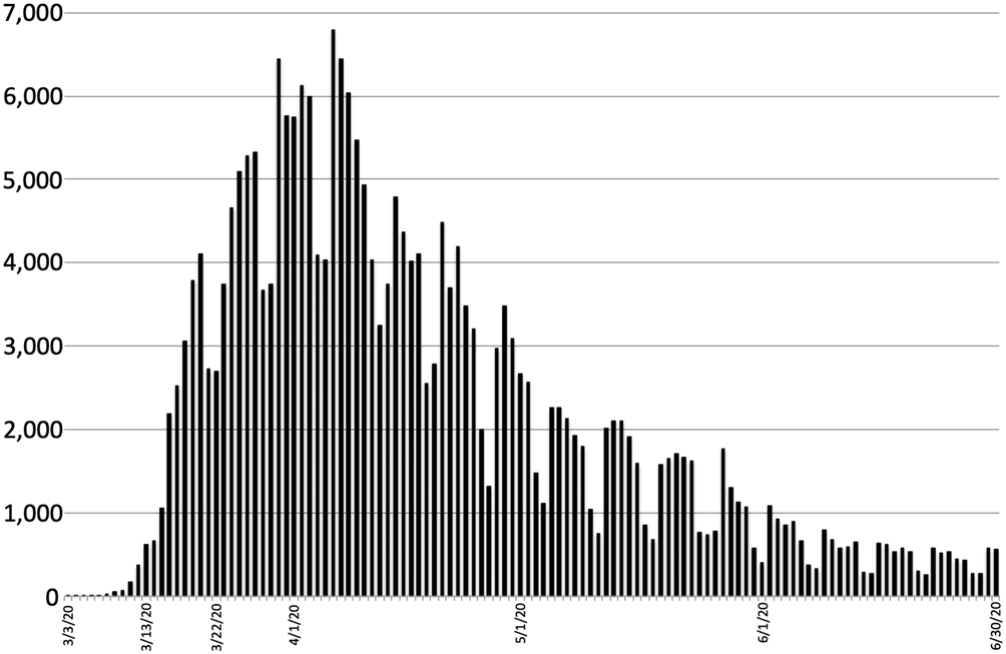
Positive COVID-19 tests in New York City by date.

On March 3, 2020, New York City confirmed its first COVID-19 case. Less than two weeks later, on March 13, 2020, in response to the World Health Organization’s declaration of the COVID-19 global pandemic and as the number of cases in New York City increased to 626, a state of emergency was imposed, limiting the number of people permitted at social gatherings. On March 22, 2020, as confirmed COVID-19 cases in New York City swelled to 2694, the New York State Governor’s office implemented an executive order banning all non-essential social gatherings of any size and forcing the complete closure of all non-essential businesses statewide (Fig. 1). Beyond the alarming global spread of the disease itself, the COVID-19 pandemic has resulted in far-reaching consequences. Unsurprisingly, early explorations into the impact of the COVID-19 pandemic on maternal populations have reported increases in anxiety and depression^24–28^. While these findings represent valuable contributions to the literature, the majority of these studies have suffered from important methodological limitations, including relying solely on self-selection recruitment^24^, social media advertisements^25,26^, unsolicited e-mails^27^ and random phone-call appeals^28^. Methods that are prone to biased sampling, may directly influence outcome measures, and are well-understood threats to the generalizability of the findings.

How the consequences of the COVID-19 pandemic lockdown may differentially impact postpartum mood among those living across the socioeconomic spectrum, both in high and low SES, who are also universally screened as part of their regular clinical care remains unknown. Therefore, the aims of this study were two-fold. The first aim was to determine any changes in postpartum mood symptomatology among patients who had given birth before and during the COVID-19 pandemic. The second aim was to explore whether there were any differences in reported postpartum mood symptomatology between those living in high and low SES during these same periods. The findings from this study will help to better understand the impact of the COVID-19 pandemic on individuals who recently gave birth, identify any differences in postpartum mood related to SES, and determine if the COVID-19 pandemic restrictions may reveal any areas for maternal health policy development^29^.

## Methods

### Sample population

The study cohort (n = 516) consisted of the entire population of patients who appeared either in person or virtually for their postpartum appointment at one of the Mount Sinai Health System sites (Mount Sinai Hospital Obstetrics and Gynecology Associates, Mount Sinai West, Mount Sinai Queens, or a Mount Sinai Hospital Faculty Practice) between January 2, 2020, and June 30, 2020. The Mount Sinai Hospital Obstetrics and Gynecology Ambulatory Practice, Mount Sinai West practice, and Mount Sinai Queens practice are New York State public health law designated community practices. They are certified as participants of the New York State Medicaid Program and primarily serve a population that self-identifies as minority. Hispanic and African-American women who reside in the inner city comprise the vast majority of the population served (90%). The remaining 10% of patients self-identify as Asian, Caucasian, Native American, Indian, Filipino, Islander, and “unknown.” All patients who utilize the hospital-based community clinics are enrolled or “pending enrollment” in a United States (U.S.) government-funded health care plan when they first come for treatment.

The Mount Sinai Faculty Practices consists of a group of independent physicians servicing a population of higher SES individuals, 59% of whom self-identify as a minority. Patients of the Faculty Practices have commercial insurance, independently subscribe to a private insurance plan, or choose to self-pay. All experimental protocols were approved and performed in compliance with the Icahn School of Medicine at Mount Sinai (ISMMS) Program for the Protection of Human Subjects and in accordance with the Health Insurance Portability and Accountability Act (HIPAA) security rule guidelines enacted in 2003 (ISMMS IRB protocol #20-03633).

### Postpartum assessment of mood

During regular operation, as a first step in screening for depressive symptomatology, the Edinburgh Postnatal Depression Scale (EPDS;^30^) is provided to all postpartum patients in their primary language in the examination room prior to meeting with a medical provider. Beginning in 2010, the Mount Sinai Health System implemented a computerized decision support “hard stop” module^31^, programmed into the postpartum electronic health record (EHR), that requires clinicians to enter the EPDS score into the EHR before the patient’s chart can be closed and the patient released. The EPDS is a ten-item self-report instrument designed to assess symptoms associated with depression and anxiety using a scale of 0–30. It is an effective screening tool for the mood changes associated with postpartum depression^32^ and has been validated in over 36 languages with considerable stability^33^. When used as a first step in screening for mood changes associated with pregnancy or when the score is used in a retrospective study for the purpose of statistical analysis, the recommended cutoff score of ≥ 9 maintains a minimal false-positive rate, a sensitivity of 89%, and a specificity of 51–93% for mood disturbance, while a cutoff score of ≥ 12 maintains a sensitivity of 62–96% and specificity of 70–98%^30,34^. During the early days of the COVID-19 pandemic, non-emergent postpartum appointments were conducted virtually or over the telephone. Administration of the EPDS over the telephone has been demonstrated as a suitable alternative for clinical practice and for research with high reliability^35^. In these instances, the EPDS was administered verbally by a clinical social worker. Once completed, the clinician was responsible for tabulating the patient’s responses and recording the EPDS score into the EHR in the regular manner.

### Data acquisition and analysis

Electronic health information relating to the study, including the date of service and EPDS score, was downloaded directly from the Mount Sinai Hospital’s network and imported into SPSS Statistics 27.0 (IBM Corporation, 2020) for analysis.

To accomplish the study’s first aim, postpartum patients were separated into two groups, those who were administered the EPDS before New York’s March 13, 2020 ban on large gatherings and those who were administered the EPDS during restrictions. Given that the total EPDS score is calculated from ordinal data, differences in reported symptomatology were assessed using Wilcoxon–Mann–Whitney tests, a statistical test that does not require the data to follow any specific distribution and that is robust to single gross outliers. Importantly, before running these tests, the distributions of the EPDS scores between each group were confirmed equivalent.

To accomplish the study’s second aim, postpartum patients were divided into high- and low-SES groups based on their location of service. Wilcoxon–Mann–Whitney tests were again run to explore any differences in reported depressive symptomatology *between* the two SES groups prior to and following the March 13, 2020 implementation of social restrictions. Once completed, additional analyses were run to determine if any changes in depression symptomatology as measured by the EPDS occurred *within* each of the SES groups, before and after implementation of the March 13, 2020 social restrictions.

### Ethical approval

The authors assert that all procedures contributing to this work comply with the ethical standards of the relevant national and institutional committees on human experimentation and with the Helsinki Declaration of 1975, as revised in 2008. All experimental protocols were approved by the Icahn School of Medicine at Mount Sinai’s (ISMMS) Program for the Protection of Human Subjects and in accordance with the Health Insurance Portability and Accountability Act (HIPAA) security rule guidelines enacted in 2003. (ISMMS IRB Protocol #20-03633).

### Informed consent

Under the Federal Policy for the Protection of Human Subjects (45 CFR 46.116; a.k.a. the "Common Rule"), informed consent was waived by determination of the Mount Sinai School of Medicine Program for the Protection of Human Subjects.

### Consent for publication

All authors.

## Results

### Characteristics of the sample

A total of 516 patients appeared for their postpartum appointment and were screened using the EPDS between January 2, 2020 and June 30, 2020, with 418 (71.9%) reporting some change in mood “over the past two weeks” (EPDS ≥ 1). A total of 64 women (12.4%) had a score ≥ 9 suggesting possible depression, and 33 women (6.4%) had a score ≥ 12 suggesting probable depression (mean = 3.8, median = 3.0, range = 0–25, SD = 4.4). A breakdown by SES is provided in Table 1.

**Table 1:** Descriptive statistics and frequencies of Edinburgh Postnatal Depression Scale (EPDS) scores across observed time periods for the sample population.

Observation date	N	Mean	Median	Range	SD	EPDS ≥ 1	EPDS ≥ 9	EPDS ≥ 12
**EPDS scores by SES: January 2, 2020 through June 30, 2020**								
Low SES	323	3.41	2.00	0–25	4.37	250 (77.4%)	43 (13.3%)	24 (7.4%)
High SES	193	4.42	3.00	0–24	4.33	168 (87.0%)	27 (14.0%)	11 (5.7%)
**EPDS scores before and after implementation of pandemic restrictions (all subjects)**								
1/2/20–3/12/2020	264	4.50	3.00	0–25	4.55	217 (82.2%)	39 (14.8%)	19 (7.2%)
3/13/2020–6/30/2020	252	3.04	1.00	0–24	4.08	201 (79.8%)	31 (12.3%)	16 (6.3%)
**EPDS scores for patients living in low SES before and after implementation of pandemic restrictions**								
Low SES 1/2/20–3/12/20	147	4.44	3.00	0–25	4.87	115 (78.2%)	21 (14.3%)	13 (8.8%)
Low SES 3/13/20–6/30/20	176	2.55	1.00	0–16	3.71	135 (76.7%)	22 (12.5%)	11 (6.3%)
**EPDS scores for patients living in high SES before and after implementation of pandemic restrictions**								
High SES 1/2/20–3/12/20	117	4.57	3.00	0–23	4.12	101 (86.3%)	18 (15.4%)	6 (5.1%)
High SES 3/13/20–6/30/20	76	4.20	3.50	0–24	4.65	67 (88.1%)	9 (11.8%)	5 (6.6%)

The ages of patients in the Ambulatory Practices ranged from 16 to 40 years, with a mean age of 27 years. Parity in the Ambulatory Practices ranged from 1 to 10 live deliveries with 21% of patients indicating this was their first delivery. The rate of cesarean deliveries in the Ambulatory Practices was 33%.

Patients of the Faculty Practice sample ranged in ages from 19 to 48 years, with a mean age of 33 years. Parity ranged from 1 to 9 live deliveries with 10% of the patients indicating this was their first delivery. The rate of cesarean deliveries in the Faculty Practice was 44%.

### Differences in mood before and during community restrictions

After dividing all postpartum patients into two groups, those who were administered the EPDS on or before March 12, 2020 and those after, corresponding to before and during community restrictions, a Wilcoxon–Mann–Whitney test demonstrated a statistically significant decrease in reported mood symptomatology following the implementation of restrictions U(N_pre-restrictions_ = 264, N_post-restrictions_ = 252) = 25,084.5, z = − 4.90, *p* < 0.001. A breakdown by these dates is provided in Table 1.

### Differences in mood between high and low economic status patients

Of the 516 patients in the sample, 323 women were classified among the low SES group, whereas 193 were among the high SES group. After running a Wilcoxon–Mann–Whitney test, no difference in depressive symptomatology as measured by the EPDS was observed between the two SES groups prior to the March 13, 2020 implementation of social restrictions (U(N_low-SES, pre-restrictions_ = 147, N_high-SES, pre-restrictions_ = 117) = 7956.0, z = − 1.05, *p* = 0.293). However, a statistically significant change in mood symptomatology was observed between the SES groups during COVID-19 restrictions (U(N_low-SES, post-restrictions_ = 176, N_high-SES, post-restrictions_ = 76) = 4895.0, z = − 3.48, *p* < 0.001), with those living in lower SES reporting a statistically significant decrease in depression symptomatology (U(N _low-SES, pre-restrictions_ = 147, N_low-SES, post-restrictions_ = 176) = 9209.0, z = − 4.56, *p* < 0.001). Notably, patients in the high SES group demonstrated no difference in symptomatology during community restrictions (U(N_high-SES, pre-restrictions_ = 117, N_high-SES, post-restrictions_ = 76) = 4045.5, z = − 1.06, *p* = 0.288). A breakdown by date and SES is provided in Table 1.

## Discussion

Even in the best environments, the period following childbirth represents a time of heightened stress and vulnerability for most, if not all, new parents. The added effects of the COVID-19 pandemic on postpartum individuals have raised considerable concern among clinicians who treat pregnant and postpartum patients. Our findings demonstrated a differentiated response in the postpartum mood of those living in New York City during the COVID-19 pandemic based on socioeconomic status. Specifically, while those in higher SES demonstrated no change in postpartum mood in light of the implementation of social restrictions in New York, those living in lower SES expressed improved mood over the same time period.

On March 13, 2020, following the World Health Organization’s declaration of the COVID-19 global pandemic two days earlier, New York City officials implemented restrictions on large social gatherings in the attempt to curb the continued spread of the virus. Shortly thereafter, on March 22, 2020, as confirmed coronavirus cases continued to increase at an alarming rate, community-wide restrictions on all social gatherings of any size and for any reason resulted in the statewide closing of all schools and non-essential business. Except for essential workers, 100% of the New York City workforce was ordered to work from home. Interestingly, while the vast majority of attention has been focused on the negative impacts and consequences of these social and economic mandates, recent observations have begun to note that the imposed social restrictions may have also had unanticipated positive effects on health and well-being^36,37^. Indeed, the well-understood social and economic factors that disproportionately impact mothers living in low SES, such as unavailable childcare, limited partner and family support, and reduced time flexibility secondary to formal and informal employment obligations, which are well understood to play a role in contributing to poorer maternal mental health^38,39^, have in many cases been ameliorated in light of these imposed restrictions. Indeed, given that those living in lower SES are understood to be at increased risk for postpartum mood dysregulation^38,39^, our finding that postpartum mood improved for those patients living in lower SES in light of the pandemic restrictions appears to have some important implications for future public health policy improvement. Specifically, health policy aimed at easing the early maternal work-child-family balance, thereby enhancing the *quality of life* for pregnant and postpartum patients living in lower SES.

It is hardly surprising that philosophers have discussed *quality of life* factors associated with human well-being that underlie physical and emotional health for millennia^40^. These differ from *standards of living* which are considered necessities for a healthy life (housing, food, education etc.). Although constituting subjective and objective measures respectively, both have been recent targets of health policy designed to mitigate the adverse effects of urban economic poverty on family mental health, often in the hopes of breaking the poverty cycle^41^. To this point, an increasing body of research has consistently found that stressors of parenting that can be buffered by enhanced institutional support, such as parental leave, result in decreased stress, increased happiness and facilitate the strengthening of the parent–child bond^42^, particularly for those living in lower SES^43^. Given that maternal mental health is directly related to the long-term physical and mental health of the offspring^44^ and that the temporary implementation of social restrictions related to the COVID-19 pandemic improved the postpartum mental health outcomes of a population understood to be at increased risk, further emphasizes the need to develop meaningful social policies to address the parental burdens of those living in urban economic poverty and towards the greater effort of interrupting the poverty cycle.

Numerous studies, both natural and randomized controlled, have sought to determine the mechanism(s) underlying the relationship between economic poverty and mental health. However, to date, the causal direction remains indeterminate. Theories generally suppose one of two potential, albeit conflicting, routes: *social selection* and *social causation*^45^. The social selection hypothesis posits that individuals with psychopathology will have reduced occupational skills, lower income, and therefore a lower SES. The literature supporting this “social drift” hypothesis however is problematic, in part, because subject samples tend to be young and unable to account for a familial income effect—except for specific incidences such as when the child’s health issues reduce the parents’ ability to maintain their income. The alternative hypothesis, social causation, proposes that people living in low SES develop psychiatric disorders as a result of adversity, including volatile income, limited support, and material hardship. While the vast majority of research appears to support this latter hypothesis^45–47^, it is equally probable that the nature of this relationship is cyclic and that while living in poverty cultivates mental illness, the consequences of mental illness likely reinforce poverty. This is mirrored by the observation that increased rates of depression among new mothers in lower SES are associated with the absence of spousal financial and social support, material deprivation and subjective standing^48^ leading to their offspring being at greater risk for cognitive^49^ and emotional challenges^50–52^. If accurate, programs designed to mitigate the social determinants of postpartum mood dysregulation in those living in lower SES could presumably offer an approach towards improving maternal health.

While these findings are important, we recognize some possible limitations of the study. First, the sample population explored was a treatment-seeking clinical sample from care-based centers in New York City, and as convenience samples, may not represent the general population of socioeconomic diversity in New York City or the United States. This is a well-understood problem universal to all health registry-based studies where the outcome variables may only represent those agreeing to treatment, as opposed to the treatment capture of all postpartum patients. In this respect, despite the benefits of universal screening, we can only assess those patients who chose to travel, recognize the benefits, or had the means to virtually attend appointments—factors well understood to affect postpartum care utilization among those living in low SES^43^. Importantly, the distinction between our method of universally screening all postpartum patients in comparison to previous studies exploring maternal mood during the COVID-19 pandemic utilizing self-selection methods^24–28^ likely explains, at least in part, the differences in our findings. That is, these earlier studies not only neglected to differentiate between SES, but in addition likely suffered from well understood methodological limitations associated with sampling bias in choosing only to study individuals who independently responded to social media announcements^24,25^, e-mails^26^, personal appeal^27^ or telephone solicitation^28^.

Second, we were limited in our ability to analyze the data for demographic differences beyond SES. It is possible a subgroup of postpartum patients exist based on demographic (e.g. age, race, parity) or obstetrical (e.g. delivery route) factors who are disproportionally experiencing postpartum mood change, either increased or decreased. However, the current sample size, while considerable, would have been insufficiently powered to meaningfully identify any differences had these factors been available to use as covariates. Therefore, this will need to be further explored. Similarly, while all subjects categorized as low SES met U.S. Government eligibility requirements for healthcare by earning less than 138 percent of the U.S. federal poverty level, those categorized as higher SES likely had considerably more income variance. Unfortunately, the resolution of our data did not afford the opportunity to explore within SES differences either.

Third, it is possible that our observation was unrelated to pandemic restrictions and due to a cyclical mood artifact, such as in response to the change in seasons. To assess this possibility, we randomly generated a year of data corresponding to the dates observed (1/2/2015–6/30/2015). We then ran similar Wilcoxon-Mann–Whitney analyses exploring for such a trend—no difference in mood was observed (See supplement 1), further supporting the pandemic restrictions as the modifier of postpartum mood improvement in lower SES patients.

Finally, the full socioeconomic implications of the COVID-19 pandemic remain yet unknown, as do the longer-term consequences of the imposed social restrictions. As we presumably remain in the early stages of the crisis, it is also possible that as the pandemic continues to extend and the impacts from unemployment, housing and childcare concerns become more acute, anxiety and depression among the postpartum population may increase. Indeed, while we observed postpartum mood improvement among those living in lower SES during restrictions, this may change with their eventual lifting and the discontinuance of policies expanding access to maternal care, housing protections, government stipends, and unemployment benefits. Furthermore, with the potential permanent loss of employment and income secondary to the projected closing of many businesses following the lifting of restrictions^53^, postpartum women of lower SES could ultimately suffer far more acutely following the pandemic than those living in higher SES, who generally have a greater ability to afford the high cost of childcare^54^ and are more likely to be able to resume many of those aspects of their lives tied to better health outcomes.

In conclusion, postpartum depression is a prevalent, cross-cultural disorder with significant morbidity. The improved postpartum mood among women of low SES in the context of temporarily imposed COVID-19-related social restrictions in New York City offers an opportunity to better understand the specific health and social support needs of postpartum patients living in urban economic poverty. Given that maternal mental illness is deleterious to the offspring and that poor mental health reinforces the poverty cycle, future health policy directed towards supporting the work-family-childcare balance of women living in low SES might assist in improving these long-term outcomes and interrupt the urban economic poverty cycle.

## Supplementary Information

Supplementary Information.

## Data Availability

The datasets generated during and analyzed during the current study are not publicly available due to patient protections and institutional policy.
