# Supplementary material for: Postpartum mood among universally screened high and low socioeconomic status patients during COVID-19 social restrictions in New York City
Source: Sci Rep. 2020 Dec 24;10:22380. doi: 10.1038/s41598-020-79564-9 (PMC7759569; doi:10.1038/s41598-020-79564-9)
Supplement: Supplementary file 1 — Supplementary Information. [file 41598_2020_79564_MOESM1_ESM.docx]

Postpartum Mood Among Universally Screened High And Low Socioeconomic Status Patients During COVID-19 Social Restrictions In New York City.

**Michael E. Silverman***

Icahn School of Medicine at Mount Sinai

The Mount Sinai Hospital

Department of Psychiatry

[Michael.silverman@mssm.edu](mailto:Michael.silverman@mssm.edu)

Tel: 212-988-0313

ORCID: 0000-0002-1297-2865

Laudy Burgos

Icahn School of Medicine at Mount Sinai

laudy.burgos@mountsinai.org

Zoe I Rodriguez

Icahn School of Medicine at Mount Sinai

Zoe.Rodriguez@mountsinai.org

Omara Afzal

Icahn School of Medicine at Mount Sinai

omara.afzal@mssm.edu

Alyssa Kalishman

MICDS

alkalishman@micds.org

Francesco Callipari

Icahn School of Medicine at Mount Sinai

Francesco.Callipari@mssm.edu

Yvon Pena

Icahn School of Medicine at Mount Sinai

yvon.pena@mountsinai.org

Ruth Gabay

Icahn School of Medicine at Mount Sinai

Ruth.Gabay@mountsinai.org

Holly Loudon

Icahn School of Medicine at Mount Sinai

holly.loudon@mssm.edu

Supplement: EPDS scores for patients living in low SES, January 2 – June 30, 2015

| **Observation Date** | **N** | **Mean** | **Median** | **Range** | **SDev** | **EPDS >1** | **EPDS >9** | **EPDS>12** |
| --- | --- | --- | --- | --- | --- | --- | --- | --- |
| 1/2/15 - 3/12/2015 | 122 | 3.47 | 3.00 | 0-17 | 3.65 | 86  (70.5%) | 13  (10.7%) | 5  (4.1%) |
| 3/13/15-6/30/15 | 177 | 4.03 | 3.00 | 0-21 | 4.48 | 128  (72.3%) | 23  (13.0%) | 16  (9.0%) |

*A Wilcoxon-Mann-Whitney test indicated no difference in EPDS scores between those patients screened on or before 3/12/2015 and those screened after (U=10010.0, z=-0.76, P=.446).
